# Research on the influence mechanism of privacy invasion experiences with privacy protection intentions in social media contexts: Regulatory focus as the moderator

**DOI:** 10.3389/fpsyg.2022.1031592

**Published:** 2023-01-10

**Authors:** Subai Chen, Chenyu Gu, Juan Wei, Mingjie Lv

**Affiliations:** ^1^School of Journalism and Communication, Xiamen University, Xiamen, China; ^2^Research Center for Intelligent Society and Social Governance, Interdisciplinary Research Institute, Zhejiang Lab, Hangzhou, China

**Keywords:** social media, privacy invasion, privacy protection, regulatory focus, privacy fatigue

## Abstract

**Introduction:**

In recent years, there have been numerous online privacy violation incidents caused by the leakage of personal information of social media users, yet there seems to be a tendency for users to burn out when it comes to privacy protection, which leads to more privacy invasions and forms a vicious circle. Few studies have examined the impact of social media users' privacy invasion experiences on their privacy protection intention. Protection motivation theory has often been applied to privacy protection research. However, it has been suggested that the theory could be improved by introducing individual emotional factors, and empirical research in this area is lacking.

**Methods:**

To fill these gaps, the current study constructs a moderated chain mediation model based on protection motivation theory and regulatory focus theory, and introduces privacy fatigue as an emotional variable.

**Results and discussion:**

An analysis of a sample of 4800 from China finds that: (1) Social media users' previous privacy invasion experiences can increase their privacy protection intention. This process is mediated by response costs and privacy fatigue. (2) Privacy fatigue plays a masking effect, i.e., increased privacy invasion experiences and response costs will raise individuals' privacy fatigue, and the feeling of privacy fatigue significantly reduces individuals' willingness to protect their privacy. (3) Promotion-focus individuals are less likely to experience privacy fatigue than those with prevention-focus. In summary, this trend of “lie flat” on social media users' privacy protection is caused by the key factor of “privacy fatigue”, and the psychological trait of regulatory focus can be used to interfere with the development of privacy fatigue. This study extends the scope of research on privacy protection and regulatory focus theory, refines the theory of protection motivation, and expands the empirical study of privacy fatigue; the findings also inform the practical governance of social network privacy.

## 1. Introduction

Nowadays, people communicate and share information through SNS, and it has become an integral part of the daily lives of network users worldwide (Hsu et al., 2013). SNS makes people's lives highly convenient. However, it also poses an increasingly serious privacy issue. For instance, British media reported that 87,000,000 Facebook users' profiles were illegally leaked to a political consulting firm, Cambridge Analytica (Revell, 2019). In addition, one of the three major US credit bureaus, Equifax, reported a large-scale data leak in 2017, including 146 million pieces of personal information (Zhou and Schaub, 2018). The incidents that happened in recent years provoked a wave of discussion on personal privacy and information security issues.

Individuals' proactive behavior in protecting online privacy information is an effective method for reducing the occurrence of privacy violations; therefore, scholars explored how to enhance individuals' willingness to protect privacy. In terms of applied theoretical models, the Health Belief Model (HBM) (Kisekka and Giboney, 2018), the Technology Threat Avoidance Theory (TTAT) (McLeod and Dolezel, 2022), the Technology Acceptance Model (TAM) (Baby and Kannammal, 2020), and the Theory of Planned Behavior (TPB) (Xu et al., 2013) have been applied to explore the issue of online privacy protection behavior. By contrast, Protection Motivation Theory (PMT) is more applicable to studying privacy protection behavior in SNS because it focuses on threat assessment and coping mechanisms for privacy issues. However, the issue with this study's application of PMT theory is that it ignores the influence of individual emotions on protective behavior (Mousavi et al., 2020). Therefore, this study considered privacy fatigue as a variable to expand the theory of PMT in the context of social media privacy protection research. Moreover, in terms of the antecedents of privacy protection, existing research suggests that factors such as perceived benefits, perceived risks (Price et al., 2005), privacy concerns (Youn and Kim, 2019), self-efficacy (Baruh et al., 2017), and trust (Wang et al., 2017) can affect individuals' privacy-protective behaviors.

Along with the increased frequency of data breaches on the Internet, people find that they have less control over their data. Further, they are overwhelmed by having to protect their privacy alone. Moreover, the complexity of the measures required to protect personal information aggravates users' sense of futility, leading to exhaustion among online users. This phenomenon, defined as “privacy fatigue,” is regarded as a factor leading to the avoidance of privacy issues. Privacy fatigue has recently been prevalent among network users. However, empirical studies related to this phenomenon are still insufficient (Choi et al., 2018). Therefore, this study attempted to explore the role privacy burnout plays in users' privacy protection behaviors. Previous studies discovered that the impact of varying degrees of privacy invasion on privacy protection differed according to individual differences. It could be moderated by psychological differences (Lai and Hui, 2006). Clarifying the role of psychological traits is beneficial to the hierarchical governance of privacy protection. Regulatory focus is a kind of psychological trait based on different regulatory orientations, which could effectively affect social media users' behavioral preferences and decisions on privacy protection (Cho et al., 2019); however, to date, the relationship between regulatory focus, privacy fatigue, and privacy protection intentions has not been sufficiently examined. For this reason, it is necessary to empirically explore this question.

Based on the PMT theoretical framework, this study built a moderated mediation model to examine the influential mechanism of privacy-invasive experiences on privacy protection intentions by introducing three factors: response costs, privacy burnout, and regulatory focus. Data analyzed from an online survey of 4,800 network users demonstrated that, first, social media users' experiences of privacy invasion increase their willingness to protect privacy. Second, privacy burnout has a masking effect, which means that the more privacy-invasive experiences and response costs there are, the greater the privacy fatigue, which reduces users' privacy protection intentions even further. Third, promotion-focused individuals are less likely to experience fatigue from protecting personal information alone. The significance of this study lies in the fact that it bridged the gap between the effects of privacy violation experiences on individuals' protective willingness.

Meanwhile, this study verified the practicality of combining PMT theory with emotionally related variables. Additionally, it complemented the study on privacy fatigue and expanded the scope of regulatory orientation theory in privacy research. From a practical perspective, this study offered a reference for the hierarchical governance of privacy in social networks. Finally, this study reveals a vicious cycle mechanism (negative experiences, privacy fatigue, low willingness to protect, and new negative experiences) followed by a theoretical reference for breaking this cycle.

## 2. Theoretical framework

### 2.1. Privacy invasion experiences, response costs, and privacy protection intentions

Protection motivation theory (PMT) is commonly used in online privacy studies (Chen et al., 2015). According to Rogers (1975), individuals cognitively evaluate the risk before adopting behaviors, develop protection motivation, and eventually modify their behaviors to avoid risks. There are two sources of impact on people's response assessments: environmental and interpersonal sources of information and prior experience. After combing through the past literature, we found that many scholars have verified the influence of environmental (Wu et al., 2019) and interpersonal (Hsu et al., 2013) factors on individual privacy protection; however, only a few scholars explored the effect of privacy violation experiences on privacy protection intentions. Some studies proved that individuals' prior privacy violation experiences are an antecedent to their information privacy concerns, including in the mobile context and at the online marketplace (Pavlou and Gefen, 2005; Belanger and Crossler, 2019). Regarding privacy concerns, prior studies widely demonstrated a significant antecedent to privacy protection intentions and protective behaviors. In addition, a meta-analysis found that users who worried about privacy were less likely to use internet services and were more likely to adopt privacy-protective actions (Baruh et al., 2017).

People make sense of the world based on their prior experiences (Floyd et al., 2000), while network users who have had privacy-invasive experiences tend to believe that the privacy risks are closely related to themselves (Li, 2008). They tend to be more aware of the seriousness and vulnerability of privacy issues (Mohamed and Ahmad, 2012). The effects of previous negative experiences on perceived vulnerability can also be explained by the availability heuristic, which assumes that the easier it is to retrieve experienced cases from memory, the higher the perceived frequency of the event. In contrast, when fewer cases are retrieved, people may estimate that the event is less likely to occur than in objective situations. Therefore, people's accumulated experiences of negative events might influence their perception of future vulnerability to risk (Tversky and Kahneman, 1974). However, in accordance with PMT, seriousness and vulnerability affect protective behavior in the context of social media privacy issues. Therefore, we can assume that the more memories of privacy violations people have, the more likely they are to believe that their privacy will be violated by privacy exposure, thereby increasing their motivation to protect privacy that is, their willingness to protect privacy. Therefore, this study proposed the following hypothesis:

**H1:** Privacy invasion experience is positively affecting protective privacy willingness.

PMT suggests that cognitive evaluation consists of assessing response costs (Rogers, 1975), and response costs refer to any costs, such as monetary, time, and effort (Floyd et al., 2000). According to findings from a health psychology study, when faced with the threat of skin cancer, people prefer to use sunscreen rather than avoid the sun (Jones and Leary, 1994; Wichstrom, 1994). It may be because of the lower response costs of utilizing sunscreen. These findings inspire us to believe that individuals calculate the response cost before they take protective actions. Privacy protection-related studies also indicate that prior experiences with personal information violations may significantly increase consumers' privacy concerns about both offline and online privacy and that privacy concerns are related to perceived risks (Okazaki et al., 2009; Bansal et al., 2010). It has also been shown that individuals who have experienced privacy invasion perceive a greater severity of risk (Petronio, 2002). Considering individuals' perceptions of risks affects their assessment of costs, which is part of the game between risks and benefits. In other words, a stronger risk perception indicates that higher response costs should be paid. Thus, this study assumed that people with more privacy violation experiences might perceive higher response costs and tend to take protective actions to avoid paying more. Consequently, this study made the following hypothesis:

**H2a:** A higher level of privacy-invasive experiences results in a higher perception of response costs.
**H2b:** A higher level of perception of response costs will result in higher privacy protection intentions.
**H2c:** Response cost mediates the effect of privacy-invasive experiences on privacy protection intentions.

### 2.2. Privacy invasion experiences, response costs, and privacy protection intentions

The medical community first introduced the concept of fatigue and referred to it as a subjective unpleasant feeling of tiredness (Piper et al., 1987). The concept of fatigue has been used in many research fields, such as clinical medicine (Mao et al., 2018), psychology, and more (Ong et al., 2006). In recent years, scholars also used the concept of “fatigue” in the study of social media and regarded it as an important antecedent to individual behaviors (Ravindran et al., 2014). Choi et al. (2018) defined “privacy fatigue” as a psychological state of fatigue caused by privacy issues. Specifically, “privacy fatigue” manifests itself as an unwillingness to actively manage and protect one's personal information and privacy (Hargittai and Marwick, 2016).

With the increasing severity of social network and personal information issues, the research around privacy fatigue, especially the examination of the antecedents and effects of privacy fatigue, has been widely developed. Regarding antecedents, scholars found that privacy concerns, self-disclosure, learning about privacy statements and information security, and the complexity of privacy protection practices could influence individuals' levels of privacy fatigue (Dhir et al., 2019; Oh et al., 2019). In terms of the effects, privacy fatigue can not only cause people to reduce the frequency of using social media or even withdraw from the Internet (Ravindran et al., 2014), but it can also motivate individuals to resist disclosing personal information (Keith et al., 2014); however, only a few studies examined privacy invasion experiences, privacy fatigue, and privacy protection intentions under one theoretical framework.

Furnell and Thomson (2009) pointed out that “privacy fatigue” is triggered by an individual's experience with privacy problems. Additionally, privacy fatigue has a boundary. When this boundary is crossed, social network users become bored with privacy management, leading them to abandon social network services. It has also been suggested that privacy data breaches can cause individuals to feel “disappointed.” In a study of medical data protection, the results showed that breaches of patients' medical data can have a cumulative effect on patients' behavioral decisions by causing them to perceive that their requests for privacy protection are being ignored (Juhee and Eric, 2018). The relationship between privacy invasion experiences and privacy fatigue has been widely demonstrated. Such social media characteristics as internet privacy threat experience and privacy invasion could lead to users' sense of emotional exhaustion and privacy cynicism, which was further associated with social media privacy fatigue (Xiao and Mou, 2019; Sheng et al., 2022). In terms of the outcomes, some other studies focusing on the privacy paradox found that emotional exhaustion and powerlessness (the same concept as exhaustion) would weaken the positive influence relationship between privacy concerns and their willingness to protect personal information (Tian et al., 2022). On account of the above reviews, it is reasonable to analogize that an individual's privacy invasion experience in the context of social media use can exacerbate an individual's perception of privacy fatigue. In other words, considering the social media privacy context, privacy fatigue may lead network users to abandon privacy protection behaviors and create opportunities for privacy invasion. Based on the above discussions, we proposed the following hypotheses:

**H3a:** Privacy invasion experiences positively affect privacy fatigue.
**H3b:** Privacy fatigue negatively affects privacy protection intentions.
**H3c:** Privacy fatigue has a masking (a form of mediating effect) role in the effects of individual social media privacy invasion experiences on privacy protection intentions.

As discussed above, we hypothesized that both response costs and privacy fatigue mediate the effect of social media users' privacy invasion experiences on their privacy protection intentions. Assuming that both response costs and privacy fatigue could mediate the effect of social media users' privacy invasion experiences on their privacy protection intentions, what is the association between response costs and privacy fatigue? It has been argued that a common shortcoming of current research applying PMT theory is that it ignores the role emotions play in this mechanism (Mousavi et al., 2020). This view is supported by Li's research, which argues that most research on privacy topics is conducted from a risk assessment perspective and tends to ignore the impact of emotions on privacy protection behaviors (Li et al., 2016). It was believed that emotions could change an individual's attention and beliefs (Friestad and Thorson, 1985). These factors are both related to behavioral intentions.

It has also been suggested that emotions play a mediating role in the process of behavioral decision-making (Tanner et al., 1991). However, only a few studies explored this influential mechanism to date. Zhang et al. (2022) found a positive influence between response costs and privacy fatigue. They conducted the research based on the Stressor-Strain-Outcome (S-S-O) framework to explore which factors (stressors) could cause privacy fatigue intentions (strain) and related behaviors (outcome). The results discovered that time cost and several other stressors significantly positively impact social media fatigue intention. As quoted from Floyd et al. (2000), “response costs” refer to any costs in which time costs were included. Despite an important reference to the above study's results provided for this study, the time cost is just one factor among response costs. This piece of research will focus on general response costs, assisting in a better understanding of this influential mechanism. Based on this, we proposed the following hypotheses:

**H4a:** Privacy response costs are positively associated with privacy fatigue.
**H4b:** Response costs and privacy fatigue play chain mediating roles in the effect of privacy invasion experiences on privacy protection intentions.

### 2.3. Regulatory focus as the moderator

Differences in individual psychological traits can lead to significant differences in individuals' cognition and behaviors (Benbasat and Dexter, 1982), and it has been shown that personal psychological traits can influence individuals' perceptions of fatigue (Dhir et al., 2019). A recent study also found that neuroticism has positive effects on privacy fatigue but that traits like agreeableness and extraversion have negative effects (Tang et al., 2021). However, previous research on social media privacy fatigue is relatively limited. Given the critical nature of privacy fatigue in research models, it is necessary to explore the differences in perceived fatigue among individuals with different psychological traits. This study introduced individual levels of regulatory focus as a moderator and examined the effect of privacy invasion experiences on privacy fatigue. Regulatory focus as a psychological trait was applied to explain social media users' privacy management and privacy protection problems (Wirtz and Lwin, 2009; Li et al., 2019).

Regulatory Focus Theory (RFT) classifies individuals into two different levels based on psychological traits: promotion focus, which focuses more on benefits and ignores potential risks, and prevention focus, which tends to avoid risks and ignore benefits when making decisions (Higgins, 1997). Research demonstrated that perceptions of benefits are supposed to reduce fatigue, while perceptions of risk could exacerbate fatigue (Boksem and Tops, 2008). By the same analogy, promotion-focused individuals are more inclined to notice the benefits of using social media (Jin, 2012) and thus may experience less fatigue and lower response costs when experiencing privacy violations; in contrast, individuals with a prevention focus are more aware of the risks associated with privacy invasion and thus have more concerns about privacy issues, which can lead to more feelings of fatigue and higher perceived response costs about privacy issues. Combined with H4, we can reason that the path of influence of social media privacy invasion experiences on privacy protection intentions may be affected by the level of individual regulatory focus. The effect of privacy invasion experiences on privacy fatigue and response costs was stronger for individuals who tended to be prevention focused than for those who tended to be promotion focused. Therefore, the mediating effect of privacy fatigue and response cost is stronger. In summary, this study proposed the hypotheses as follows:

**H5a:** Compared to promotion-focused users, the effect of privacy invasion experiences on privacy fatigue is greater for prevention-focused users.
**H5b:** Compared to prevention-focused users, the effect of privacy invasion experiences on response costs is greater for promotion-focused users.

### 2.4. Current study

**Figure 1:**
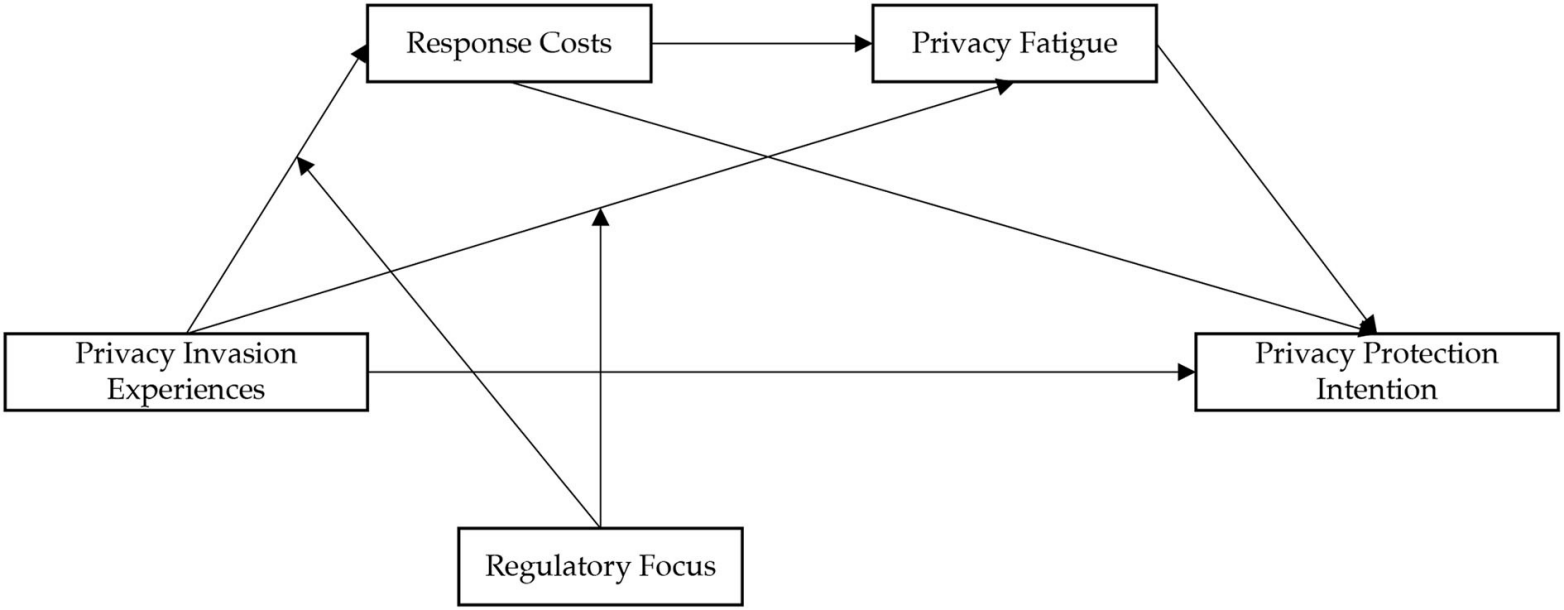
Conceptual model.

In summary, the current study concluded that, in the social media context, users' experiences of privacy invasion would increase their perception of response costs and thus result in privacy fatigue. Privacy fatigue decreases individuals' privacy protection intentions. However, this process differed for individuals with different regulatory focuses. In detail, individuals with a promotion focus are less likely to experience privacy fatigue than individuals with a prevention focus. Based on the above logic, the conceptual model constructed in this study is shown in Figure 1.

## 3. Materials and methods

### 3.1. Participants and procedures

This survey was conducted in December 2021, and Zhejiang Lab collected the data. The questionnaire was pretested with a small group of participants to ensure the questions were clearly phrased. Participants were informed of their right to withdraw and were assured of confidentiality and anonymity before participating in this research survey. Computers, tablets, and mobile phones were all used to complete the cross-sectional survey. After giving their consent, participants were asked to complete the following scales. After the screening, 4,800 valid questionnaires were selected. The invalid questionnaires were deleted mainly based on not passing the test of the screening questions rather than not answering the questions carefully (e.g., the answers to the questions of several consecutive variables are the same, or the number of repeated options is >70%).

To guarantee data quality and reduce possible interference from gender and geographical factors, this survey used a quota sampling method, as shown in Table 1, with a sample gender ratio of 1:1 and samples from 16 cities in China, with 300 valid samples in each city. Considering the possible relationship between the privacy invasion experience and the years of Internet usage, participants' previous privacy invasion experience is meaningful to this study, and the final sample had 34.5 and 57.3% of Internet usage between 5 and 10 years and more than 10 years, respectively, which met the requirements of the study. In terms of education level, college and bachelor's degrees accounted for the largest proportion, at 62.0%, followed by high school/junior high school and vocational high school, at 27.3%. In terms of the age of the sample, the ratio of those younger than 46 years old to those above was 59.7:40.3 with a balanced distribution among all age groups. The basic demographic variables are tabulated as shown in Table 1.

**Table 1:** Statistical table of basic information on effective samples.

**Statistical items**	**Specific content**	**Statistical value**	**Percentage/%**
Gender	Men	24,00	50.0
	Women	24,00	50.0
Age	18~25	357	7.4
	26~35	1,573	32.8
	36~45	936	19.4
	Over 46	1,934	40.3
Educational background	Under High School	356	7.4
	High School	1,308	27.3
	Undergraduate	2,975	62.0
	Master and Doctor	161	3.4
Internet life time	Less than 3 years	34	0.7
	3~5 years	356	7.4
	5~10 years	1,658	34.5
	Over 10 years	2,752	57.3

### 3.2. Measurements

Based on the model and hypotheses of this study, the instruments of this study included measures of privacy invasion experiences, response costs, privacy fatigue, privacy protection intentions, and regulatory focus (including promotion focus and prevention focus). This study's questionnaire was designed on scales that have been pre-validated. All scales were adapted based on social media contexts, and all responses were graded on a Likert scale ranging from 0 (strongly disagree) to 6 (strongly agree). A higher score was a better fit for that measure. Sub-items within each scale were averaged, resulting in composite scores.

The privacy invasion experiences scale was referenced from Su's study (Su et al., 2018). The scale is a 3-item self-reported scale (e.g., “My personal information, such as my phone number, shopping history, and more, is used to be shared by intelligent media with third-party platforms.”). The response cost scale was developed from the scale in the study by Yoon et al. (2012), which included three measurement questions (e.g., “When personal information security is at risk on social media, I consider that taking practical action will take too much time and effort.”). The privacy fatigue scale was derived from a related study by Choi et al. (2018), and the current study applied this 4-item scale to measure privacy fatigue on social media (e.g., “Dealing with personal information protection issues on social media makes me tired.”). The privacy protection intention scale was based on the scale developed by Liang and Xue (2010), which contains three measurement items (e.g., “When my personal information security is threatened on social media, I am willing to make efforts to protect it.”). The regulatory focus scale was derived from the original scale developed by Higgins (2002) and later adapted by Chinese scholars for use with Chinese samples (Cui et al., 2014). The scale contains six items on measures for promotion focus (e.g., “For what I want to do, I can do it all well”) and four items on measures for prevention focus (e.g., “While growing up, I often did things that my parents didn't agree were right”). The regulatory focus was measured by subtracting the average prevention score from the average promotion score, with higher differences indicating a greater tendency toward promotion focus and lower differences indicating a greater tendency toward prevention focus (Cui et al., 2014).

### 3.3. Data analysis

The validity and reliability of our questionnaire were tested using Mplus8. The PROCESS macro for SPSS was used to evaluate the moderated chain mediation model with the bootstrapping method (95 percent CI, 5,000 samples). Gender (1 = men, 0 = women), age, the highest degree obtained, and Internet lifetime are among the covariates examined in this model.

## 4. Results

### 4.1. Measurement of the model

As shown in Table 2, privacy invasion experiences, response costs, privacy fatigue, and privacy protection intentions are all factors to consider. Cronbach's α and composite reliability of scales are higher than the acceptable value (>0.70). Although the Cronbach's α for promotion and prevention focus were slightly <0.70, they were >0.60 and close to 0.70, which was also considered permissible due to the large sample size of this study, and the reliability test of the measurement model in this study was qualified (Hair et al., 2019).

**Table 2:** Results of the validity and reliability.

	**1**	**2**	**3**	**4**	**5**	**6**	**AVE**	**CR**	**Cronbach's α**
1. PIE	**0.729**						0.724	0.773	0.767
2. RC	0.468	**0.825**					0.594	0.862	0.862
3. PF	0.457	0.538	**0.773**				0.784	0.857	0.856
4. PPI	0.106	0.075	−0.153	**0.654**			0.518	0.751	0.750
5. Pro Focus	0.051	0.020	−0.093	0.451	**0.655**		0.420	0.683	0.693
6. Pre Focus	0.338	0.287	0.449	−0.030	−0.002	**0.668**	0.442	0.703	0.697

PIE, privacy invasion experiences; PC, response costs; PF, privacy fatigue; PPI, privacy protection intentions. Bold value is the square root of AVE.

Since the measurement instruments in this study were derived from validated scales, the average variance extracted (AVE) was higher than 0.5, but we can accept 0.4. According to Fornell and Larcker (1981), if the AVE is <0.5, but the composite reliability is higher than 0.6, the construct's convergent validity is still acceptable (Fornell and Larcker, 1981). Further, Lam (2012) also explained and confirmed this view (Lam, 2012). Discriminant validity was tested by comparing the square root of AVE with the correlations of the researched variables. The square root of the AVE was higher than the correlation, indicating good discriminant validity.

Then, we tested the goodness of fit indices. Confirmatory factor analysis (CFA) of our questionnaire produced acceptable fit values for the one-dimensional factor structure (RMSEA = 0.048 0.15, SRMR = 0.042 0.05, GFI = 0.955 > 0.9, CFI = 0.947 > 0.9, NFI = 0.943 > 0.9, and 948 = 0.945 > 0.9) after introducing the error covariances in the model. In summary, the current study passed the reliability and validity tests.

### 4.2. Descriptive statistics

Table 3 shows the descriptive statistics and correlation analysis results. Response costs, privacy fatigue, and privacy protection intentions were all positively correlated with privacy invasion experiences. Privacy fatigue and privacy protection intentions were both positively correlated with response costs. Private fatigue was found to be negatively related to privacy protection intentions.

**Table 3:** Means, standard deviations, and correlations among research variables.

**Research variables**	***M***	**SD**	**1**	**2**	**3**	**4**	**5**
1. PIE	3.525	1.304	1				
2. RC	3.797	1.441	0.468^**^	1			
3. PF	2.807	1.477	0.457^**^	0.538^**^	1		
4. PPI	4.636	0.882	0.106^**^	0.075^**^	−0.153^**^	1	
5. RF	1.637	1.476	−0.265^**^	−0.239^**^	−0.440^**^	0.271^**^	1

PIE, privacy invasion experiences; PC, response costs; PF, privacy fatigue; PPI, privacy protection intentions; RF, regulatory focus; ^**^p < 0.01.

### 4.3. Relationship between privacy invasion experience and privacy protection intentions

Table 4 shows the results of the polynomial regression analysis. Privacy invasion experiences significantly influenced levels of response costs (β = 0.466, SE = 0.023, *t* = 11.936, *p* = 0.000), privacy fatigue (β = 0.297, SE = 0.022, *t* = 13.722, *p* = 0.000), and privacy protection intentions (β = 0.133, SE = 0.011, *t* = 12.382, *p* = 0.000) after controlling for gender, highest degree obtained, age, and Internet lifetime. Response costs positively predicted privacy fatigue (β = 0.382, SE = 0.013, *t* = 29.793, *p* = 0.000) and privacy protection intention (β = 0.098, SE = 0.010, *t* = 9.495, *p* = 0.000). However, privacy fatigue was significantly negatively correlated with privacy protection intentions (β = −0.130, SE = 0.011, *t* = −12.303, *p* = 0.000) in this model. In conclusion, H1, H2a, H2b, H3a, H3b, and H4a were supported.

**Table 4:** Multiple regression results of the moderated mediation model.

**Independent variable**	**β**	**SE**	***T***	***P***	***R^2^***	***F***
**Dependent variable: privacy protection intentions (PIE)**				
PIE	0.133	0.011	12.382	0.000^***^	0.134	106.295^***^
PF	−0.130	0.011	−12.303	0.000^***^		
RC	0.098	0.010	9.495	0.000^***^		
**Dependent variable: privacy fatigue (PF)**				
PIE	0.297	0.022	13.722	0.000^***^	0.427	446.246^***^
RC	0.382	0.013	29.793	0.000^***^		
RF	−0.101	0.040	−2.510	0.0121^*^		
PIE × RF	−0.031	0.008	−4.103	0.000^***^		
**Dependent variable: response costs (RC)**				
PIE	0.466	0.023	11.936	0.000^***^	0.234	209.354^***^
RF	−0.143	0.046	−3.138	0.0017^**^		
PIE × RF	0.007	0.009	0.840	0.401		

PIE, privacy invasion experiences; PC, response costs; PF, privacy fatigue; PPI, privacy protection intentions; RF, regulatory focus; ^*^p < 0.05; ^**^p < 0.01; ^***^p < 0.001; β, unstandardized regression weight; SE, standard error for the unstandardized regression weight; t, t-test statistic; F, F-test statistic.

Then, we used Model 6 of PROCESS to test the mediating effect in our model. As the results in Table 5, H2c, H3c, and H4b were accepted.

**Table 5:** Results of mediating effect test.

	**Path**	**Effect**	**95% Boot CI**	

			**LLCI**	**ULCI**
Indirect effect	PIE → RC → PPI	0.053	0.041	0.065
	PIE → PF → PPI	−0.057	−0.065	−0.049
	PIE → RC → PF → PPI	−0.042	−0.048	−0.037
Total indirect effect		−0.047	−0.059	−0.035

PIE, privacy invasion experiences; PC, response costs; PF, privacy fatigue; PPI, privacy protection intentions.

Model 84 in the SPSS PROCESS macro is applied to carry out the bootstrapping test to examine the moderation effect of regulatory focus. Privacy invasion experiences, response costs, privacy fatigue, and regulatory focus were centralized before constructing the interaction term. The results showed that regulatory focus significantly moderated the effect of privacy invasion experiences on privacy fatigue [95% Boot CI = (0.002, 0.006), and H5a was supported. In addition, the mediating effect was significant at a low level of regulatory focus (−1 SD; Effec*t* = −0.038; 95% Boot CI = (−0.046, −0.030)], medium level of regulatory focus [Effec*t* = −0.032; 95% Boot CI = (−0.039, −0.026)] and high level of regulatory focus [+1 SD; Effec*t* = −0.026; 95% Boot CI = (−0.032, 0.020)]. Specifically, the mediating effect of privacy fatigue decreased as individuals increasingly tended to be promotion focused. However, the regulatory focus did not significantly moderate the effect of privacy invasion experiences on response costs [95% Boot CI = (−0.001, 0.003)], and H5b was rejected.

**Figure 2:**
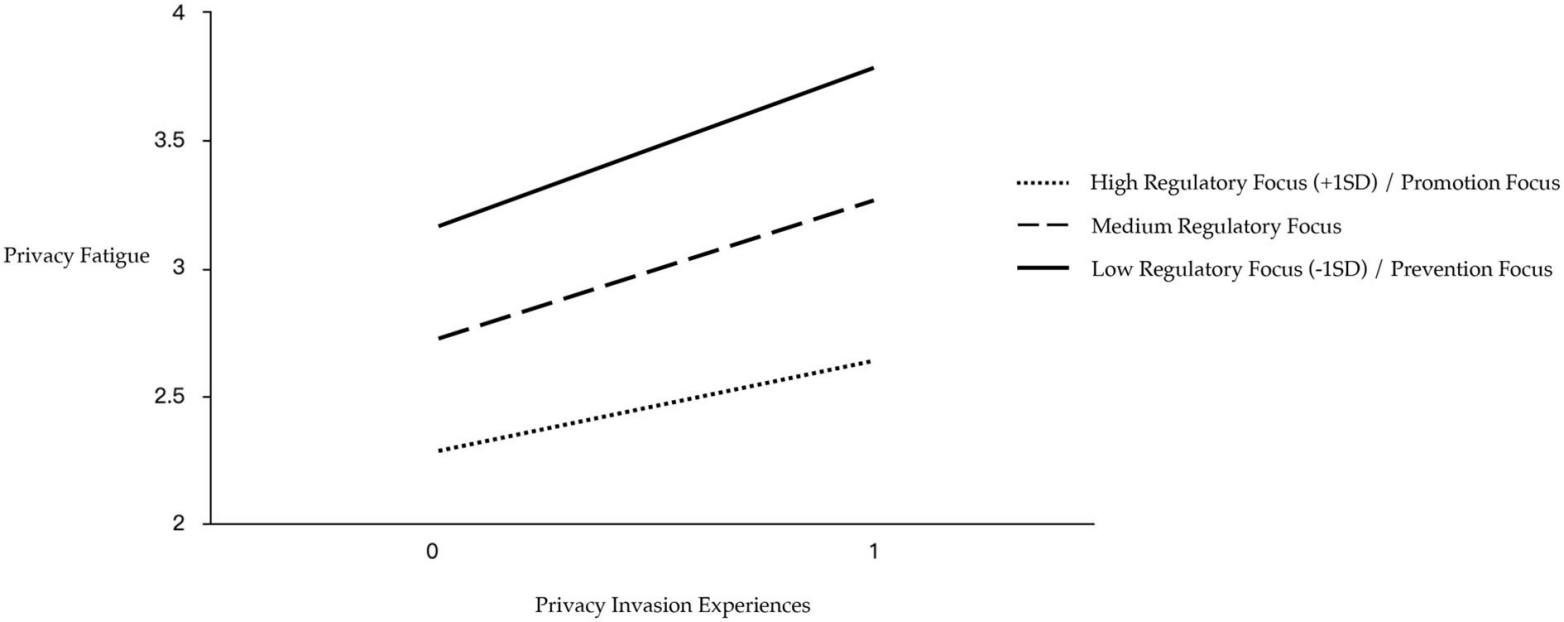
Simple slope test of the interaction between PIE and RF on the PF.

Meanwhile, privacy invasion experiences × regulatory focus interaction significantly predicted privacy fatigue (β = −0.046, SE = 0.008, *t* = −3.694, *p* = 0.000; see Figure 2). The influence of privacy invasion experiences on privacy fatigue was significant when the level of regulatory focus was high (β = 0.385, SE = 0.016, *t* = 23.981, *p* = 0.000), medium (β = 0.430, SE = 0.015, *t* = 29.415, *p* = 0.000), and low (β = 0.475, SE = 0.022, *t* = 22.061, *p* = 0.000). Specifically, the more the individuals tended to be promotion focused (high regulatory focus scores), the less the level of fatigue caused by privacy invasion, and the more the individuals tended to be prevention focused (low regulatory focus scores), the more the level of fatigue was caused by privacy invasion.

## 5. Discussion

**Figure 3:**
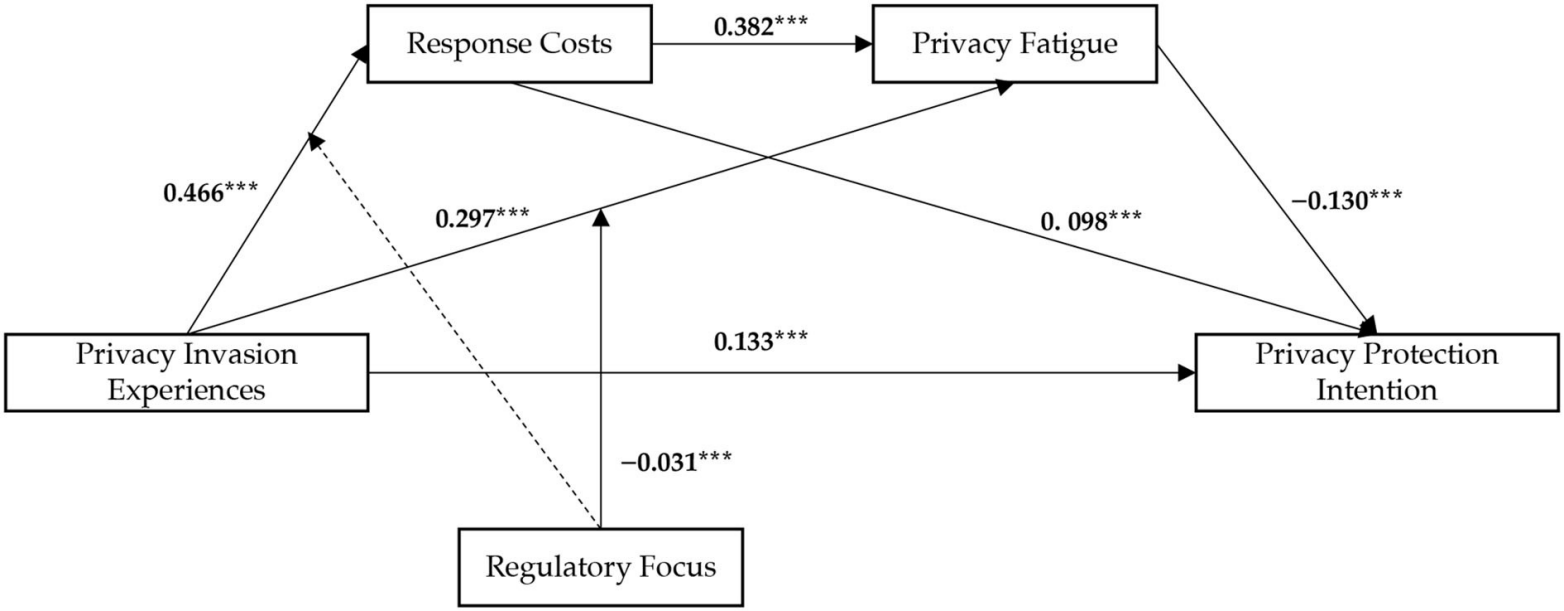
The moderated chain mediation model. Dashed lines represent nonsignificant relations ^***^p < 0.001.

The purpose of the present study was to explore the relationship among privacy invasion experiences, response costs, privacy fatigue, privacy protection intentions, and regulatory focus. This study showed that response costs and privacy fatigue play mediating roles, whereas regulatory focus plays a moderating role in this process (as shown in Figure 3). These findings help clarify how and under which circumstances social media users' privacy invasion experiences affect their privacy protection intentions, thereby providing a means to improve people's privacy situation on social media platforms.

### 5.1. A chain mediation of response costs and privacy fatigue

The current study found that social media users' privacy invasion experiences have a significant positive effect on their response costs, and the increase in response costs will in turn increase individuals' privacy protection intentions. This finding was consistent with previous literature on health psychology, which found that individuals calculate response costs for different actions before making decisions. The higher the response costs individuals perceive, the greater the possibility that they will improve their protective intention (Jones and Leary, 1994; Wichstrom, 1994). Compared with users who experienced less privacy invasion on social media, people who experienced more privacy violations would perceive a higher level of response costs, which would further increase their protective intention to avoid dealing with the negative outcomes followed by privacy invasion.

The study also found that social media users' privacy invasion experiences had a significant positive effect on privacy fatigue, which is consistent with prior research on social media use (Xiao and Mou, 2019; Sheng et al., 2022). At the same time, response costs also positively affected privacy fatigue, and research on social media fatigue behaviors indicated this influential mechanism in the past (Zhang et al., 2022). However, this study additionally found that response costs partially mediated the effect of privacy invasion experiences on privacy fatigue. Although both increased privacy invasion experiences and increased response costs will improve social media users' privacy protection intentions, privacy fatigue can mask this process, i.e., increased privacy fatigue reduces individuals' privacy protection intentions.

Moreover, this study revealed that response costs and privacy fatigue play chain-mediated roles in the effect of social media privacy invasion experiences on privacy protection intentions and further explained the mechanism. In addition, the masking effect of privacy fatigue also explains why privacy invasion experiences do not have a strong effect on privacy protection intentions. In other words, this privacy fatigue is an important reason that people currently “lie flat” (adopt passive protection) in the face of privacy-invasive issues online.

### 5.2. Regulatory focus as moderator

The relationship between social media privacy invasion experiences and privacy fatigue was moderated by regulatory focus. To be more specific, the more the people who promoted their privacy, the less the level of privacy fatigue they felt; the more the people who prevented their privacy, the more the level of privacy fatigue they felt. In other words, promotion focus acts as a buffer in this process. In other words, promotion focus has a buffering effect in this process. To some extent, the result of this study verified that different regulated individuals would sense different levels of fatigue due to their pursuing benefits or avoiding risks when they make decisions (Boksem and Tops, 2008; Jin, 2012). On the other hand, the regulatory focus did not moderate the relationship between privacy invasion experiences and response costs. One possible explanation is that, compared with privacy fatigue, response costs to privacy violations are based on exact experiences in users' memories. Individuals who have had more privacy invasions have more experience dealing with the negative consequences of privacy violations. Thus, whether psychological traits were added or not, the effect of privacy-invasive experiences on response costs would not be strengthened or weakened.

Meanwhile, this study has proven a moderated mediation model investigating the moderating role of regulatory focus in mediating “privacy invasion experiences—privacy fatigue—privacy protection intentions.” The results indicated that, as individuals tend to be prevention focused, privacy invasion experiences affect individuals' privacy protection intentions through the mediating role of privacy fatigue; specifically, the more they tend to be prevention focused, the stronger their privacy fatigue and the weaker their privacy protection intentions. Therefore, interventions for privacy fatigue (e.g., improving media literacy, creating a better online environment, and more) can be used to enhance social media users' privacy protection intentions (Bucher et al., 2013; Agozie and Kaya, 2021). In particular, focusing on social media users who tend to be prevention focused is crucial.

### 5.3. Implication

From a theoretical perspective, our study found a mechanism for influencing privacy-protective behavior based on an extension of the protective motivation theory. Protection motivation theory is a fear-based theory. We used our experiences with social media privacy invasions as a source of fear. Based on this, we found that these experiences were associated with individuals' privacy protection intentions. We explained the mechanism through the mediating variable of response costs, which is also consistent with previous findings (Chen et al., 2016).

More importantly, however, in response to what previous researchers have argued is an emotional factor that traditional protection motivation theory ignores (Mousavi et al., 2020), our study extended traditional protection motivation theory to include privacy fatigue as a factor and verified that fatigue significantly reduces social media users' privacy protection intentions. The introduction of “privacy fatigue” can better explain why occasional privacy invasion experiences do not cause privacy-protective behaviors, which is another possible explanation for the privacy paradox in addition to the traditional privacy calculus theory. The introduction of “privacy fatigue” has also inspired researchers to pay attention to individual emotions in privacy research. This study also compared differences in privacy protection intentions among social media users of different regulatory focus types, which are mainly caused by fatigue rather than response costs. By combining privacy fatigue and regulatory focus, it was found that not all subjects felt the same level of privacy fatigue after experiencing privacy invasion. This study also expanded the application of both privacy fatigue and regulatory focus theories and built a bridge between online privacy research and regulatory focus theory.

In addition to the aforementioned implications for research and theory, the findings also have some useful, practical implications. First of all, the findings of this piece ask for measures to reduce privacy invasion on social media. (a) Reducing the incidence of privacy violations at their root requires improving the current online privacy environment on social media platforms. We call on the government to strengthen the regulation of online privacy and social media platforms to reinforce the protection of users' privacy. To a large extent, users' personal information should not be misused. (b) From the social media agent perspective, relevant studies mentioned that content relevance perceived by online users could mitigate the negative relations between privacy invasion and continuous use intention (Zhu and Chang, 2016). Social media agents should improve their efficiency in using qualified personal information, giving users a smoother experience on online platforms.

Second, the results show that privacy fatigue could affect users' privacy protection intentions. (c) According to Choi et al. (2018), users have a tolerance threshold for privacy fatigue. The policy should formulate an acceptable level of privacy protection. Other scholars suggested that online service providers should avoid excessively or unnecessarily collecting personal information and forbid sharing or selling users' personal information strictly with any third party without their permission (Tang et al., 2021). (d) Another effective way is to reduce response costs to reduce the costs of protecting one's privacy. For example, social media platforms can optimize privacy interfaces and management tools or provide more effective feedback mechanisms for users. (e) In addition, improving users' privacy literacy (especially for prevention-focused individuals) can also be effective in reducing privacy fatigue (Bucher et al., 2013).

Finally, different measures should be applied based on different regulatory-focused users. (f) Social media managers could further classify users into groups based on their psychological characteristics and manage them in accordance with their requirements for the level of privacy protection. Thereby, social media users may have a wider range of choices. Specifically, due to previous privacy invasive experience, prevention-focused individuals tend to feel more privacy fatigue, requiring additional privacy protection features for prevention-focused users. For example, social media platforms could offer specific explanations of privacy protection technologies to increase prevention-focused individuals' trust in privacy protection technologies.

### 5.4. Limitations and future directions

There are still some limitations present in this article. Firstly, this study solely selected response costs as individuals' cognitive process, whereas threat appraisal was also included in the cognitive process of protection motivation theory, which focused on the potential outcomes of risky behaviors, including perceived vulnerability, perceived severity of the risk, and rewards associated with risky behavior (Prentice-Dunn et al., 2009). Future studies could systematically consider the association between these factors and privacy protection intentions. Second, users' perceptions of privacy invasion are different across various social media platforms (e.g., Instagram and Facebook), and this study only applies to a generalized social media context. Future research could pay more attention to the differences among users on different social media platforms (with different functions). Finally, this study did not focus on specific privacy invasion experiences. However, studies pointed out that different types of privacy invasions affect people differently. Moreover, people with different demographical backgrounds, such as cultural backgrounds and gender, would react differently when faced with the same situation (Klein and Helweg-Larsen, 2002). Future research can investigate this in more depth through experiments.

## 6. Conclusion

In conclusion, our findings suggest that social media privacy invasion experiences increase individuals' privacy protection intentions by increasing their response costs, but e increase in privacy fatigue masks this effect. Pivacy fatigue is a barrier to increasing social media users' willingness to protect their privacy, which explains why users do not seem to show a stronger willingness to protect their privacy when privacy invasion is a growing problem in social networks nowadays. Our study also revealed a different level of fatigue that individuals with different levels of regulatory focus exhibit when faced with the same level of privacy invasion experience. In particular, prevention-focused social media users are more likely to become fatigued. Therefore, social media agents should pay special attention to these individuals because they may be particularly vulnerable to privacy violations. Furthermore, the current research on privacy fatigue has yet to be expanded, and future researchers can add to it.

Our theoretical analysis and empirical results further emphasize the distinction between individuals, a differentiation that allows researchers to align their analyses with theoretical hypotheses more tightly. This applies not only to research on the effects of privacy invasion experiences on privacy behavior but also to exploring other privacy topics. Therefore, we recommend that future privacy research be more human-oriented, which will also benefit the current “hierarchical governance” of the Internet privacy issue.

## Data availability statement

The original contributions presented in the study are included in the article/Supplementary material, further inquiries can be directed to the corresponding author.

## Ethics statement

This study was approved by the Academic Committee of the School of Journalism and Communication at Xiamen University, and we carefully verified that we complied strictly with the ethical guidelines.

## Author contributions

CG is responsible for the overall research design, thesis writing, collation of the questionnaire, and data analysis. SC and ML are responsible for the guidance. JW is responsible for the proofreading and article touch-up. All authors contributed to the article and approved the submitted version.
